# Integrative Transcriptomics Reveals a Mitochondria-Related Gene Signature in Periodontitis

**DOI:** 10.3390/biom16091369

**Published:** 2026-09-20

**Authors:** Siyuan Wu, Miaomiao Feng, Mengyao Liu, Qing Zhang, Gaixin Xu, Mengzhen Gao, Cory J. Xian, Hongli Chen, Yuankun Zhai

**Affiliations:** 1 School of Stomatology, Henan University, Kaifeng 475000, China; 2 Kaifeng Key Laboratory of Periodontal Tissue Engineering, Kaifeng 475000, China; 3 School of Pharmacy and Biomedical Sciences, College of Health, Adelaide University, Adelaide City Campus, Adelaide, SA 5005, Australia; cory.xian@adelaide.edu.au

**Keywords:** periodontitis, mitochondria, transcriptomics, machine learning, *CBR3*, *CYP24A1*, *SLC25A45*, *SSBP1*

## Abstract

Mitochondrial dysfunction contributes to periodontal inflammation and tissue destruction, yet the mitochondria-related transcriptional changes associated with periodontitis remain incompletely characterized. Using an integrative bioinformatics study design with experimental validation in cellular and animal models, we sought to identify a mitochondria-related gene signature in affected periodontal tissues and evaluate selected candidates experimentally. Differential expression analysis and weighted gene co-expression network analysis of GSE16134 were integrated with MitoCarta3.0 annotation. Candidate genes were evaluated using 101 feature-selection and classification combinations across GSE16134, GSE10334, GSE33774, and GSE173078, followed by functional enrichment, pathway-level analysis, and immune-cell deconvolution. Selected candidates were assessed by reverse-transcription quantitative polymerase chain reaction in lipopolysaccharide-stimulated RAW 264.7 macrophages and by immunohistochemistry in a ligature-induced rat periodontitis model. The integrated analysis identified 25 candidate genes. A backward stepwise generalized linear model combined with random forest selected *CBR3*, *CYP24A1*, *SLC25A45*, *SSBP1*, and *TMEM205* and achieved the highest mean area under the receiver operating characteristic curve (AUC = 0.819) across the four datasets. The corresponding AUCs were 0.995 in GSE16134, 0.894 in GSE10334, 0.661 in GSE33774, and 0.727 in GSE173078. The model achieved an AUC of 0.870 in the independent validation cohort GSE223924. The selected genes exhibited distinct expression patterns in affected gingival sites and were associated with pathways involving mitochondrial metabolism, cellular respiration, and inflammatory signaling. Immune-cell deconvolution further revealed associations between the selected genes and multiple B- and T-cell subsets. Experimental analyses consistently reproduced decreased CBR3 and increased CYP24A1 and SSBP1 expression in both the macrophage and rat models. These findings delineate a mitochondria-centered transcriptional landscape associated with periodontitis and prioritize *CBR3*, *CYP24A1*, and *SSBP1* as candidate molecular markers for further investigation. By integrating cross-dataset transcriptomic analysis with experimental evidence, this study provides a focused basis for subsequent mechanistic investigation and clinical validation of mitochondria-related candidate markers in periodontitis.

## 1. Introduction

Periodontitis is a chronic inflammatory disease arising from dysbiotic subgingival communities and a dysregulated host response, culminating in destruction of the periodontal ligament, cementum, and alveolar bone [1]. The Global Burden of Disease 2021 analysis estimated an age-standardized prevalence of approximately 12.5% for severe periodontitis, underscoring its substantial and persistent public-health burden [2]. Although dysbiosis is central to disease initiation, the severity and progression of tissue breakdown are shaped by how resident and infiltrating cells integrate immune, oxidative, metabolic, environmental, and systemic cues [1,3]. The host molecular programs that translate these convergent stress signals into persistent inflammation and periodontal tissue destruction remain incompletely defined.

Mitochondria integrate cellular bioenergetics, redox homeostasis, metabolite signaling, calcium handling, and innate immune activation [4,5]. Accordingly, mitochondrial dysfunction has increasingly been implicated in periodontal inflammation and alveolar bone loss [4,6]. Beyond adenosine triphosphate generation, mitochondria regulate reactive oxygen species (ROS) production, mitophagy, and inter-organelle communication [5]. Excessive mitochondrial ROS can damage mtDNA and respiratory-chain components, thereby sustaining a self-amplifying cycle of oxidative stress and inflammatory signaling [7]. In periodontal tissues, impaired mitochondrial quality control has been experimentally linked to osteoclast dysregulation and alveolar bone loss [8,9]. Recent transcriptomic, single-cell, and machine-learning studies have begun to identify molecular signatures linking mitochondrial dysfunction, mitophagy, and immune alterations to periodontitis [10,11,12,13,14]. Nevertheless, the reproducibility of these signatures across independent cohorts and their consistency with experimental models remain insufficiently established, leaving a gap between computational associations and experimentally supported disease-related molecular patterns.

Public transcriptomic datasets enable systematic mapping of disease-associated gene patterns across cohorts and platforms. Machine-learning methods can support feature prioritization and classification [15], but high-dimensional models remain vulnerable to overfitting, data leakage, cohort overlap, and confounding by cell composition [16,17]. These concerns are particularly relevant when multiple tissue samples originate from the same participant or when candidate signatures are advanced as biomarkers [17]. An integrative strategy that couples mitochondrial annotation and network-level biology with transparent cross-dataset assessment is therefore required to distinguish reproducible disease-related signals from cohort- and model-specific effects.

In this integrative bioinformatics and experimental study, we combined human gingival transcriptomics, weighted gene co-expression network analysis (WGCNA), MitoCarta3.0-based mitochondrial annotation, and a 101-combination machine-learning framework to identify mitochondria-related molecular features of periodontitis across four datasets. Functional enrichment and immune-cell deconvolution were used to define the biological and cellular contexts of the selected genes, and selected candidates were further examined in lipopolysaccharide-stimulated macrophages and ligature-induced rat periodontitis. By bridging multi-dataset computational discovery with experimental evaluation, this study aimed to delineate a mitochondria-centered transcriptional landscape of periodontitis and prioritize molecular candidates at the interface of mitochondrial dysfunction, periodontal inflammation, and tissue destruction.

## 2. Materials and Methods

### 2.1. Study Design and Transcriptomic Datasets

This study was designed as an integrative bioinformatics and experimental study, combining observational analyses of publicly available human gingival transcriptomic datasets with experimental validation in cellular and animal models.

Datasets were selected according to the following inclusion criteria: (1) human periodontal tissue samples; (2) a comparison between periodontitis-affected and control or reference tissues; (3) gene expression profiling by microarray or RNA sequencing; and (4) more than 10 eligible tissue samples in total, with at least five samples in each comparison group. Datasets involving non-human samples, non-periodontal tissues, no relevant comparison group, or insufficient eligible samples were excluded. Sample-size requirements were applied after excluding samples from other disease categories.

Five publicly available human gingival transcriptomic datasets were obtained from the Gene Expression Omnibus (GEO) database (https://www.ncbi.nlm.nih.gov/geo/, accessed on 1 May 2025). The datasets were analyzed separately; samples from different platforms were not pooled. GSE16134 was used for discovery and model development [18]. GSE10334, which was generated from an earlier and partially overlapping Columbia University cohort, was treated as a reproducibility dataset rather than an independent external validation cohort [18,19]. GSE33774 and GSE173078 were used as additional evaluation cohorts in the original model-selection framework [20,21]. Gingivitis samples in GSE173078 and peri-implantitis samples in GSE33774 were excluded from the periodontitis-versus-reference comparisons. GSE223924 was additionally included as an independent validation cohort; only the periodontitis and healthy gingival tissue samples were included, whereas peri-implantitis samples were excluded.

Table 1 summarizes the datasets and their analytical roles. In GSE16134 and GSE10334, the reference samples were clinically unaffected gingival sites obtained from participants with periodontitis; they therefore do not represent periodontally healthy individuals [18,19]. In contrast, GSE223924 included healthy gingival tissue samples as the reference group. Processed expression matrices and platform annotations were downloaded from GEO and analyzed at the gene-symbol level. A total of 1136 human mitochondrial genes were obtained from the MitoCarta3.0 inventory (https://www.broadinstitute.org/mitocarta/mitocarta30-inventory-mammalian-mitochondrial-proteins-and-pathways, accessed on 1 May 2025) [22].

### 2.2. Differential Expression Analysis

Differential expression analysis was performed in GSE16134 using the limma package (version 3.62.2) [23]. A gene-wise linear model was fitted to compare affected with clinically unaffected gingival sites, followed by empirical Bayes moderation of the variance estimates. Genes with |log_2_FC| > 1 and an adjusted *p* value (FDR) < 0.05 were defined as differentially expressed genes. Principal-component analysis was used to examine the overall distribution of the samples, and heatmap and volcano plots were generated to visualize differential-expression patterns.

### 2.3. Weighted Gene Co-Expression Network Analysis

Weighted gene co-expression network analysis was performed in GSE16134 using the WGCNA package (version 1.73) [24]. Genes with a Benjamini–Hochberg-adjusted *p* value < 0.05 in the initial between-site comparison were ranked according to the absolute difference in median expression between affected and clinically unaffected sites, and the top 5000 genes were included in network construction. Candidate soft-thresholding powers were evaluated using the pickSoftThreshold function, and β = 16 was selected to approximate scale-free network topology. Pairwise gene-expression correlations were transformed into an adjacency matrix, which was subsequently converted into a topological overlap matrix (TOM). Average-linkage hierarchical clustering was performed using TOM-based dissimilarity, and co-expression modules were identified using dynamic tree cutting.

Each module was summarized by its module eigengene, and Pearson correlation analysis was used to evaluate the relationships between module eigengenes and periodontal site status. The module showing the strongest positive correlation with affected sites was selected for subsequent analysis. Within this module, gene significance was calculated as the correlation between individual gene expression and site status, whereas module membership was calculated as the correlation between individual gene expression and the corresponding module eigengene.

The differentially expressed genes and genes assigned to the selected WGCNA module were separately intersected with the MitoCarta3.0 inventory. The union of the two resulting mitochondria-related gene sets was retained as the candidate gene set for subsequent functional and machine-learning analyses.

### 2.4. Functional Enrichment Analysis

Functional enrichment analysis of the 25 candidate genes was performed using the clusterProfiler package (version 4.14.6) [25]. Gene Ontology (GO) (https://geneontology.org/, accessed on 10 May 2025) enrichment was examined separately for the biological process, cellular component, and molecular function categories, and Kyoto Encyclopedia of Genes and Genomes (KEGG) (https://www.genome.jp/kegg/, accessed on 10 May 2025) pathway enrichment was analyzed using the human pathway annotation. *p* values were adjusted for multiple testing using the Benjamini–Hochberg method. GO terms and KEGG pathways were ranked according to their adjusted *p* values, and the leading annotations were visualized. An adjusted *p* value < 0.05 was considered statistically significant.

### 2.5. Machine-Learning Framework and Model Evaluation

The 25 candidate genes were used as input features in a combinatorial machine-learning framework comprising 10 algorithms: least absolute shrinkage and selection operator, ridge regression, elastic net, stepwise generalized linear modeling (Stepglm), partial least-squares regression for generalized linear models, generalized linear model boosting, random forest (RF), support vector machine, linear discriminant analysis, and naive Bayes. Single algorithms and sequential combinations of feature-selection and classification algorithms yielded 101 candidate models, following the multicohort combinatorial machine-learning strategy described previously [26].

All models were fitted using GSE16134, and their performance was assessed by receiver operating characteristic (ROC) analysis across GSE16134, GSE10334, GSE33774, and GSE173078. Candidate models were ranked according to the mean area under the ROC curve (AUC) across the four datasets. The feature set retained by the highest-ranked model was selected for subsequent analyses. Dataset-specific AUCs and corresponding 95% confidence intervals were calculated using the pROC package (version 1.19.0.1) [27]. The final model was further evaluated in an independent validation cohort (GSE223924) to assess its generalizability in an external dataset.

### 2.6. Gene-Level Expression and Pathway Analyses

The expression levels of the selected genes were compared between affected and clinically unaffected gingival sites in GSE16134 using the two-sided Wilcoxon rank-sum test. Pairwise relationships among the selected genes were evaluated using Pearson correlation analysis. Gene-specific ROC curves were constructed from continuous expression values in GSE16134, and the corresponding AUCs were calculated using the pROC package (version 1.19.0.1) [27].

Gene set enrichment analysis (GSEA) was performed using clusterProfiler (version 4.14.6) [25,28]. For each selected gene, samples were dichotomized at the median expression level into high- and low-expression groups for exploratory pathway analysis. Genes were ranked according to genome-wide log2 fold changes between the high- and low-expression groups and subjected to pathway enrichment analysis. Pathways with a nominal *p* value < 0.05, an absolute normalized enrichment score > 1, and a false-discovery rate *q* value < 0.25 were retained as exploratory pathway associations. Gene set variation analysis (GSVA) was performed using the GSVA package (version 2.0.7) [29] to calculate sample-level pathway enrichment scores from the normalized expression matrix. Pearson correlation analysis was subsequently used to evaluate the relationships between the expression of each selected gene and the corresponding GSVA pathway scores.

### 2.7. Immune-Cell Deconvolution

CIBERSORT was applied to the normalized expression matrix of GSE16134 using the LM22 reference signature and 1000 permutations to estimate the relative fractions of 22 immune-cell populations [30]. Differences in the inferred immune-cell fractions between affected and clinically unaffected gingival sites were assessed using the two-sided Wilcoxon rank-sum test. Pearson correlation analysis was used to examine the correlation structure among immune-cell fractions and the relationships between selected-gene expression and individual immune-cell populations. For the gene–immune-cell correlation analyses, *p* values were adjusted for multiple comparisons using the Benjamini–Hochberg method, with BH-adjusted *p* < 0.05 considered statistically significant.

### 2.8. Cell Culture and Inflammatory Stimulation

RAW 264.7 murine macrophage-like cells were obtained from Procell Life Science & Technology Co., Ltd. (Wuhan, China). Cells were maintained in Dulbecco’s modified Eagle’s medium (DMEM; Gibco, Thermo Fisher Scientific, Waltham, MA, USA) supplemented with 10% fetal bovine serum (Proteintech, Rosemont, IL, USA) and 1% penicillin–streptomycin (Gibco, Thermo Fisher Scientific, Waltham, MA, USA) at 37 °C in a humidified atmosphere containing 5% CO_2_. Cells in the logarithmic growth phase were used for all experiments. For inflammatory stimulation, cells were seeded in six-well plates and allowed to adhere overnight. Following serum starvation, cells were exposed to 1 μg/mL lipopolysaccharide derived from *Escherichia coli* O55 (LPS; catalog no. L2880; Sigma-Aldrich, St. Louis, MO, USA) for 24 h. Control cells underwent the same culture and medium-change procedures without LPS treatment. Cells were collected immediately after treatment for RNA extraction and gene-expression analysis.

### 2.9. RNA Extraction and RT-qPCR

Total RNA was extracted from control and LPS-treated cells using TRIzol reagent (Invitrogen, Thermo Fisher Scientific, Waltham, MA, USA) according to the manufacturer’s instructions. RNA concentration and purity were assessed using an ultra-micro spectrophotometer (Allsheng Instruments Co., Ltd., Hangzhou, China). RNA was subsequently reverse-transcribed into complementary DNA using an A200 Gradient Thermal Cycler (LongGene, Hangzhou, China). Reverse-transcription quantitative polymerase chain reaction (RT-qPCR) was performed using Universal SYBR qPCR Super MIX (catalog no. QS-D012; Qisabio, Zhengzhou, China) on a QuantStudio 3 Real-Time PCR System (Applied Biosystems, Thermo Fisher Scientific, Waltham, MA, USA). Amplification consisted of an initial denaturation step at 95 °C for 30 s, followed by 40 cycles at 95 °C for 5 s and 60 °C for 30 s. Melting-curve analysis was performed after amplification to assess product specificity [31]. Relative mRNA expression levels were calculated using the 2^−ΔΔCt^ method [32], with *GAPDH* as the internal reference gene. Primer sequences were synthesized by Sangon Biotech, Shanghai, China and are provided in Table 2.

### 2.10. Animals and Ligature-Induced Periodontitis

All animal procedures were approved by the Biomedical Research Ethics Subcommittee of Henan University (approval no. HUSOM2025-949) and are reported in accordance with the ARRIVE 2.0 guidelines [33]. Twelve male Sprague–Dawley rats aged 6–8 weeks were obtained from Henan Skbes Biotechnology Co., Ltd. (Anyang, China). Animals were housed under specific pathogen-free conditions with a 12-h light/dark cycle at 22 ± 2 °C and 50 ± 10% relative humidity. Standard laboratory diet and water were available *ad libitum*. After one week of acclimatization, animals were randomly allocated to the control and periodontitis groups (*n* = 6 per group). For periodontitis induction, rats were anesthetized with inhaled isoflurane. A 3-0 nonabsorbable suture was placed subgingivally around the cervical region of each bilateral maxillary second molar and secured on the palatal side. No ligatures were placed in control animals. Ligatures were maintained for 14 days. Gingival condition and probing pocket depth were assessed at the experimental endpoint. Body weight was monitored during the experimental period. At necropsy, the heart, liver, spleen, lungs, and kidneys were collected and weighed, and organ indices were calculated relative to terminal body weight.

At the endpoint, animals were deeply anesthetized with isoflurane and euthanized by gradual-fill CO_2_ inhalation in accordance with the approved institutional protocol. Cervical dislocation was performed after loss of consciousness as a secondary method to ensure death. Death was confirmed by the absence of respiration, heartbeat, and reflex responses before tissue collection. Maxillae and major organs were harvested for subsequent imaging and histological analyses.

### 2.11. Micro-CT Analysis

Maxillae were harvested and fixed in 4% paraformaldehyde for 24 h. Samples were scanned using a SkyScan 1276 micro-computed tomography system (Bruker, Kontich, Belgium) at 90 kV and 88 μA with an isotropic voxel size of 18 μm over a 360° rotation. Projection images were reconstructed using NRecon software (version 2.0), and the reconstructed datasets were reoriented to a consistent anatomical position using DataViewer (version 1.5.6.2). A standardized region of interest encompassing the alveolar bone surrounding the maxillary second molar was defined and analyzed using CTAn (version 1.20). Alveolar bone loss was assessed by measuring the distance from the cementoenamel junction to the alveolar bone crest (CEJ–ABC). Three-dimensional bone morphometry included bone volume fraction (BV/TV), trabecular thickness (Tb.Th), trabecular separation (Tb.Sp), and trabecular number (Tb.N). All morphometric measurements were independently performed by two trained operators who were blinded to the experimental group allocation. Before analysis, both operators completed technical training for the SkyScan 1276 micro-CT system. Measurements obtained by the two operators were averaged for subsequent statistical analysis.

### 2.12. Histology and Immunohistochemistry

Maxillary specimens were decalcified in 10% EDTA at room temperature for 4 weeks, dehydrated through a graded ethanol series, cleared in xylene, and embedded in paraffin. Mesiodistal sections containing the maxillary second molar were prepared at a thickness of 4 μm. Serial sections were stained with hematoxylin and eosin or Masson’s trichrome according to standard procedures. Major-organ sections were stained with hematoxylin and eosin. All stained sections were scanned using an EasyScan6 slide scanner (Maccura Biotechnology Co., Ltd., Chengdu, China).

For immunohistochemistry, maxillary sections were deparaffinized and rehydrated, followed by trypsin-mediated antigen retrieval at 37 °C for 20 min. Endogenous peroxidase activity was blocked with 3% hydrogen peroxide for 30 min, after which nonspecific binding was blocked with serum. Sections were incubated overnight at 4 °C with rabbit polyclonal primary antibodies against CBR3 (Proteintech, 15619-1-AP, 1:100), CYP24A1 (Proteintech, 21582-1-AP, 1:100), and SSBP1 (Proteintech, 12212-1-AP, 1:100). After three washes with phosphate-buffered saline, sections were incubated with the appropriate secondary antibodies at 37 °C for 15 min. Immunoreactivity was visualized using diaminobenzidine, and nuclei were counterstained with Mayer’s hematoxylin. Immunostained sections were scanned using the EasyScan6 system. The DAB-positive area within a standardized periodontal region of interest was quantified using ImageJ (version 1.54) and expressed as a percentage of the total tissue area analyzed. The same image-acquisition and analysis settings were applied to all groups. Histological assessment and immunohistochemical quantification were independently performed by two trained assessors blinded to the experimental group allocation. Both assessors had completed technical training for the EasyScan6 slide-scanning system and the corresponding image-analysis workflow. Measurements obtained by the two assessors were averaged for subsequent analysis.

### 2.13. Statistical Analysis

Bioinformatics analyses were performed using R version 4.4.1, and Venn diagrams were generated using an online bioinformatics platform (http://www.bioinformatics.com.cn, accessed on 1 June 2025). Experimental data were analyzed using GraphPad Prism version 9.0. For cell experiments, the independent biological experiment was treated as the statistical unit, with technical replicates averaged before analysis. For animal experiments, the individual animal was treated as the statistical unit. For image-based morphometric, histological, and immunohistochemical measurements assessed by two blinded observers, the mean of the two independent measurements was used for statistical analysis. Two-group comparisons were performed using the two-sided Wilcoxon rank-sum test. Data are presented as the mean ± standard deviation. Receiver operating characteristic performance is reported as the area under the curve with corresponding 95% confidence intervals. Benjamini–Hochberg correction was applied where specified for multiple testing. All statistical tests were two-sided, and *p* < 0.05 was considered statistically significant.

## 3. Results

### 3.1. Identification of Periodontitis- and Mitochondria-Associated Genes

**Figure 1:**
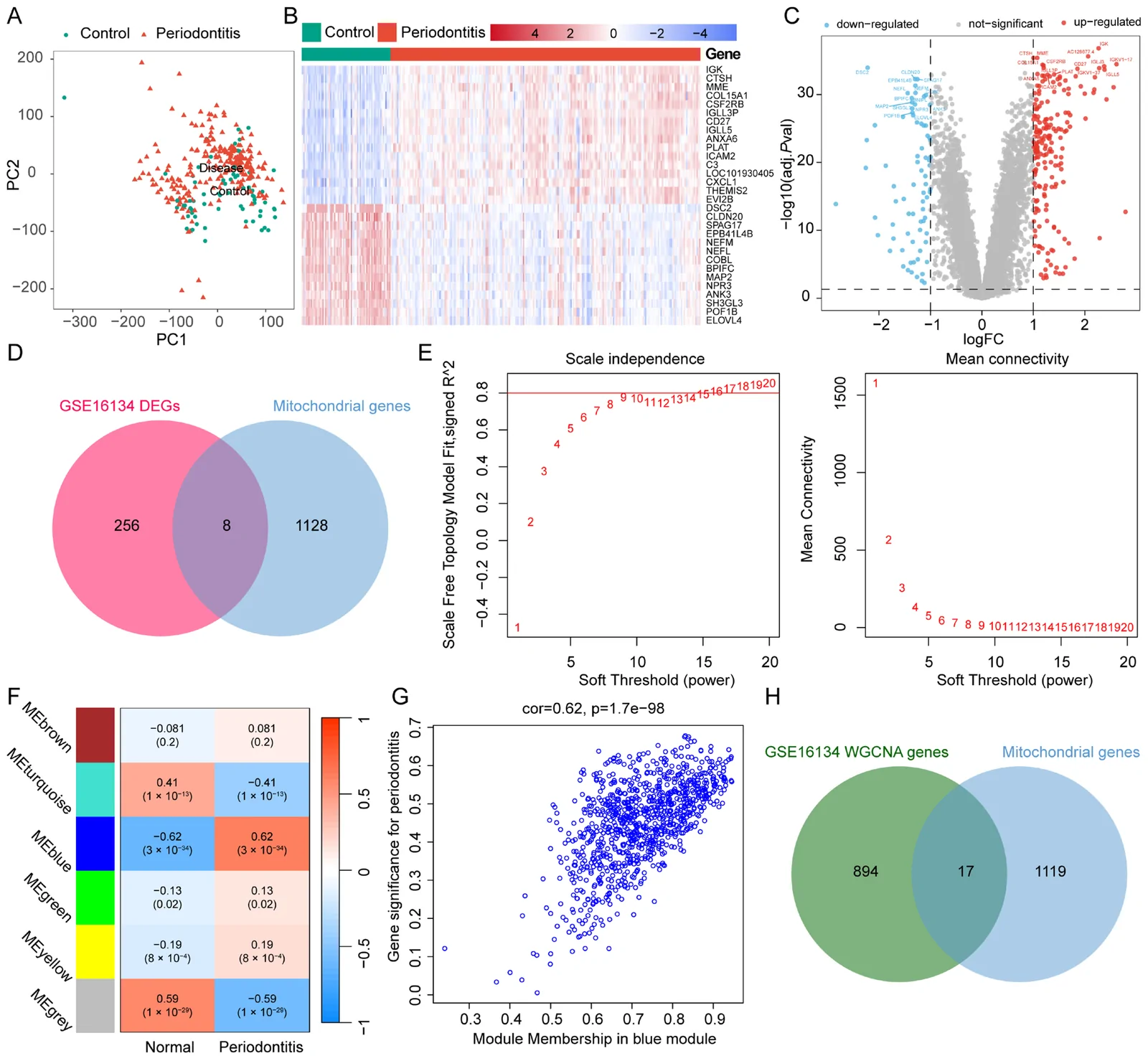
Identification of mitochondria-related candidate genes associated with periodontal site status. (**A**) Principal-component analysis of GSE16134. (**B**) Heatmap and (**C**) volcano plot showing differentially expressed genes between affected and clinically unaffected gingival sites. (**D**) Intersection of the differentially expressed genes with the MitoCarta3.0 inventory. (**E**) Selection of the soft-thresholding power for weighted gene co-expression network analysis (β = 16). (**F**) Heatmap of correlations between module eigengenes and periodontal site status. (**G**) Relationship between module membership and gene significance in the blue module. (**H**) Intersection of genes assigned to the blue module with the MitoCarta3.0 inventory. WGCNA, weighted gene co-expression network analysis.

GSE16134 comprised 310 gingival-site samples obtained from 120 patients with periodontitis. Principal-component analysis revealed partial separation between affected and clinically unaffected sites, although the two groups remained substantially overlapping (Figure 1A). Differential expression analysis identified 264 genes, of which 188 showed higher expression and 76 showed lower expression in affected sites (Figure 1B,C). Eight of these differentially expressed genes were included in the MitoCarta3.0 inventory (Figure 1D).

For weighted gene co-expression network analysis, a soft-thresholding power of β = 16 was selected to approximate scale-free topology (Figure 1E). Five co-expression modules were identified, together with an unassigned grey gene set. Among these modules, the blue module showed the strongest positive correlation with affected periodontal sites (*r* = 0.62, *p* = 3.0 × 10^−34^; Figure 1F). Within this module, module membership was positively correlated with gene significance (*r* = 0.62, *p* = 1.7 × 10^−98^; Figure 1G). Seventeen of the 911 genes assigned to the blue module were included in MitoCarta3.0 (Figure 1H). The union of the 8 differentially expressed mitochondria-related genes and the 17 WGCNA-derived mitochondria-related genes yielded 25 candidate genes for subsequent analyses.

### 3.2. Functional Annotation of the Candidate Genes

**Figure 2:**
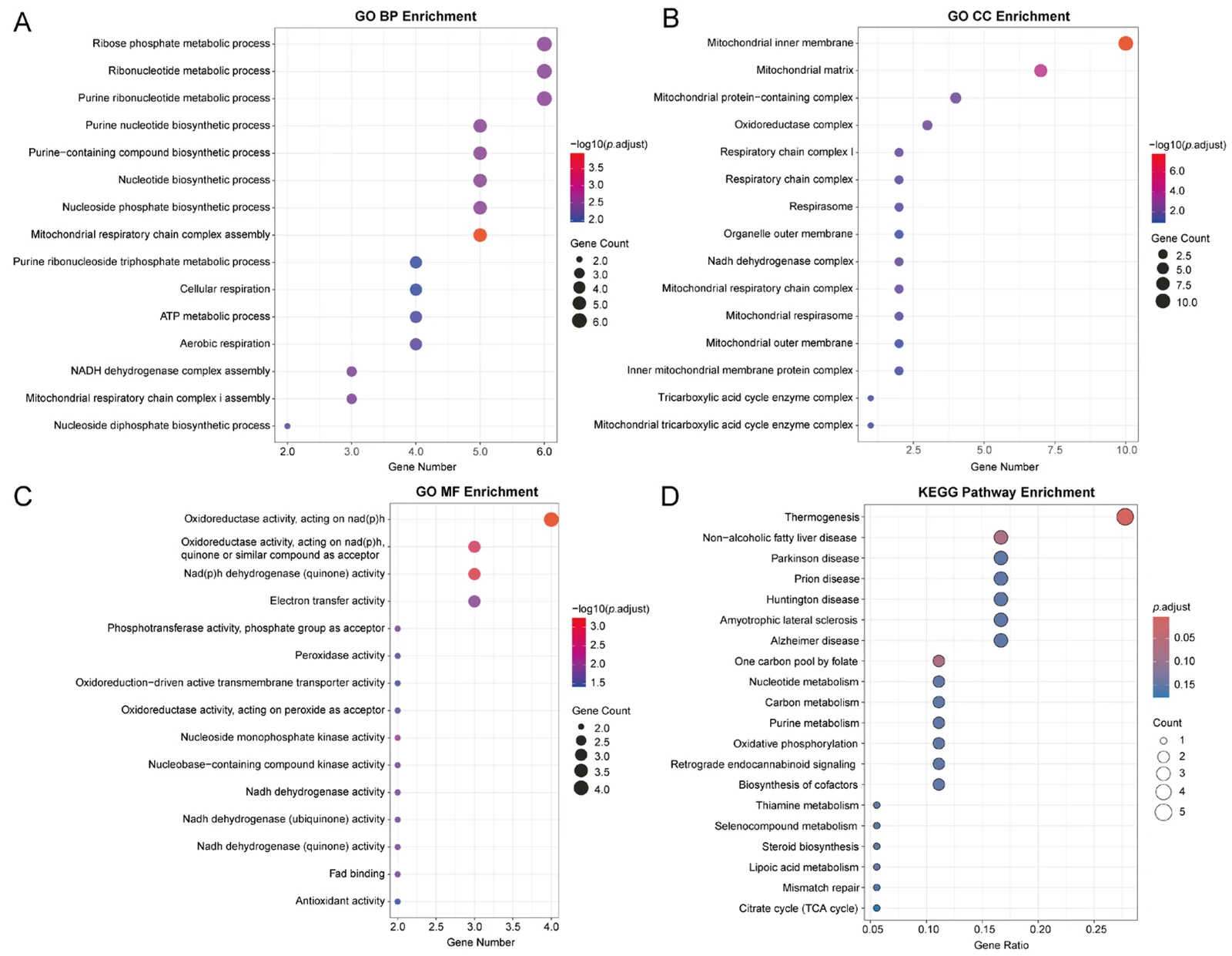
Functional annotation of the 25 mitochondria-related candidate genes. Gene Ontology enrichment analyses of (**A**) biological processes, (**B**) cellular components, and (**C**) molecular functions. (**D**) Kyoto Encyclopedia of Genes and Genomes pathway analysis. Color represents the Benjamini–Hochberg-adjusted *p* value, and point size represents the number of genes assigned to each term. GO, Gene Ontology; KEGG, Kyoto Encyclopedia of Genes and Genomes.

Gene Ontology analysis showed that the 25 candidate genes were significantly associated with ribose-phosphate, ribonucleotide, purine-nucleotide, and ATP metabolic processes, as well as cellular respiration and mitochondrial respiratory-chain complex assembly (Figure 2A). Cellular-component annotations were dominated by the mitochondrial inner membrane, mitochondrial matrix, respiratory-chain complexes, and oxidoreductase complexes (Figure 2B). The principal molecular-function terms included NAD(P)H-dependent oxidoreductase activity, NADH dehydrogenase activity, and electron-transfer activity (Figure 2C). Thermogenesis was the top-ranked KEGG pathway. Other leading annotations included oxidative phosphorylation, nucleotide, purine, carbon, one-carbon, and tricarboxylic-acid-cycle metabolism, together with several mitochondria-related disease pathway annotations (Figure 2D). Complete GO enrichment analyses of all 264 DEGs and all 911 genes in the WGCNA blue module (Figure S1; Tables S1 and S2) showed predominant enrichment in immune- and inflammation-related terms (e.g., leukocyte migration, B-cell activation, and antigen receptor signaling), providing the broader functional context within which the mitochondria-related subsets are embedded.

### 3.3. Machine-Learning Selection and Cross-Dataset Performance

**Figure 3:**
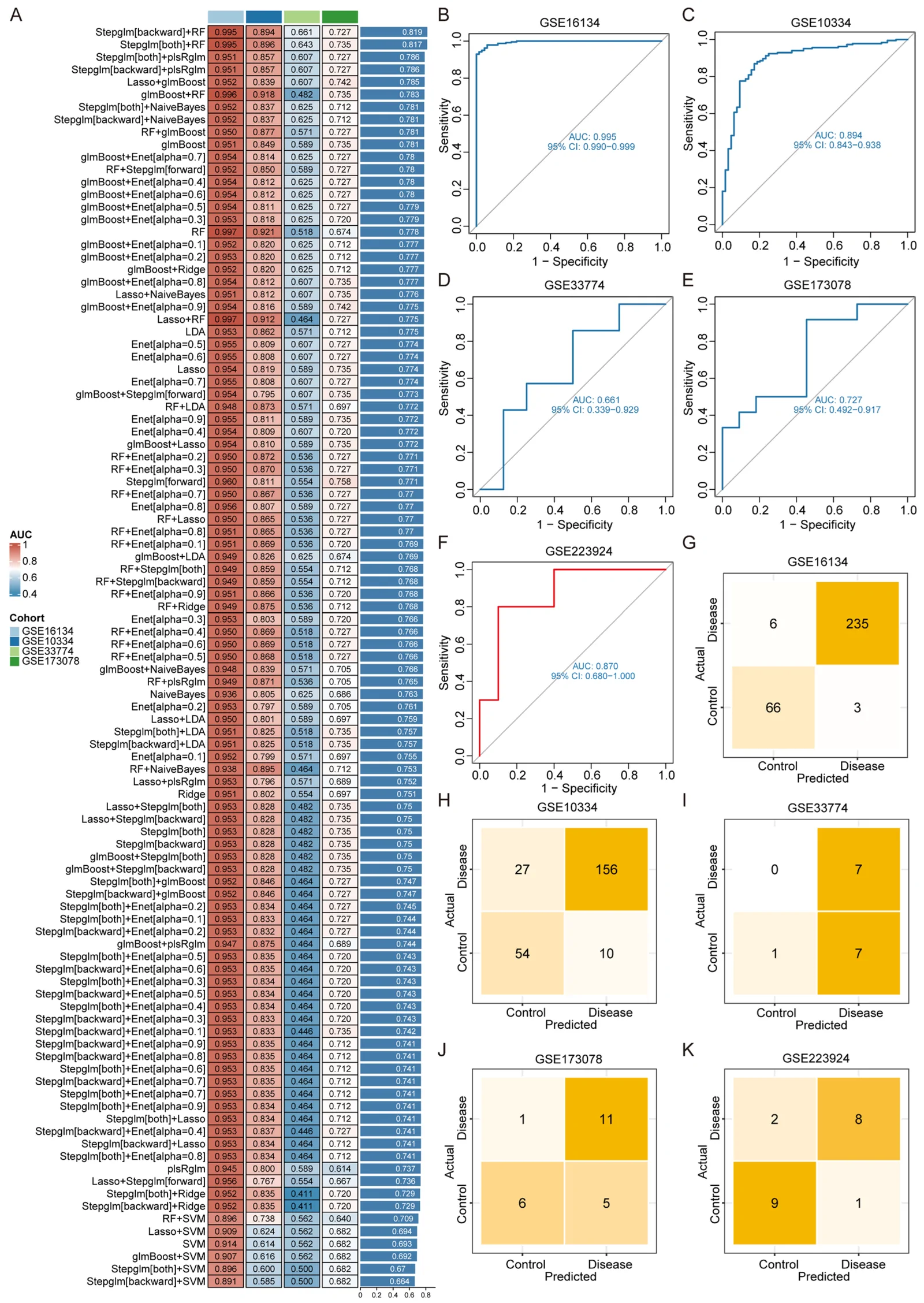
Machine-learning model selection and performance across datasets. (**A**) Area under the receiver operating characteristic curve for 101 algorithmic combinations evaluated in GSE16134, GSE10334, GSE33774, and GSE173078. The final column indicates the mean AUC across the four datasets. (**B**–**F**) ROC curves for the Stepglm[backward] + RF model in GSE16134, GSE10334, GSE33774, GSE173078, and the independent validation cohort GSE223924, respectively. The red ROC curve in (**F**) represents the independent validation cohort (GSE223924), whereas the blue ROC curves in (**B**–**E**) represent the discovery and evaluation cohorts. The gray diagonal lines indicate the reference line for random classification. (**G**–**K**) Confusion matrices showing the classification performance of the Stepglm[backward] + RF model in GSE16134, GSE10334, GSE33774, GSE173078, and GSE223924, respectively. Color intensity in the confusion matrices reflects the number of samples in each cell. AUC, area under the curve; CI, confidence interval; RF, random forest; ROC, receiver operating characteristic; Stepglm, stepwise generalized linear model.

The 25 candidate genes were evaluated using 101 machine-learning models. The Stepglm[backward] + RF combination produced the highest mean AUC across the four datasets (mean AUC = 0.819; Figure 3A). Its site-level AUC was 0.995 (95% CI, 0.990–0.999) in GSE16134, 0.894 (95% CI, 0.843–0.938) in GSE10334, 0.661 (95% CI, 0.339–0.929) in GSE33774, and 0.727 (95% CI, 0.492–0.917) in GSE173078 (Figure 3B–E). In the independent validation cohort GSE223924, the model achieved an AUC of 0.870 (95% CI, 0.680–1.000; Figure 3F). Confusion matrices for the five datasets are shown in Figure 3G–K, and the corresponding diagnostic performance metrics are summarized in Table 3. Calibration curves for the five cohorts are shown in Figure S2, demonstrating relatively good calibration in the discovery cohort GSE16134 and greater deviations from the ideal line in some cohorts with smaller sample sizes. The highest-ranked model retained a five-gene feature set comprising *CBR3*, *CYP24A1*, *SLC25A45*, *SSBP1*, and *TMEM205*.

### 3.4. Expression and Pathway Associations of the Selected Genes

**Figure 4:**
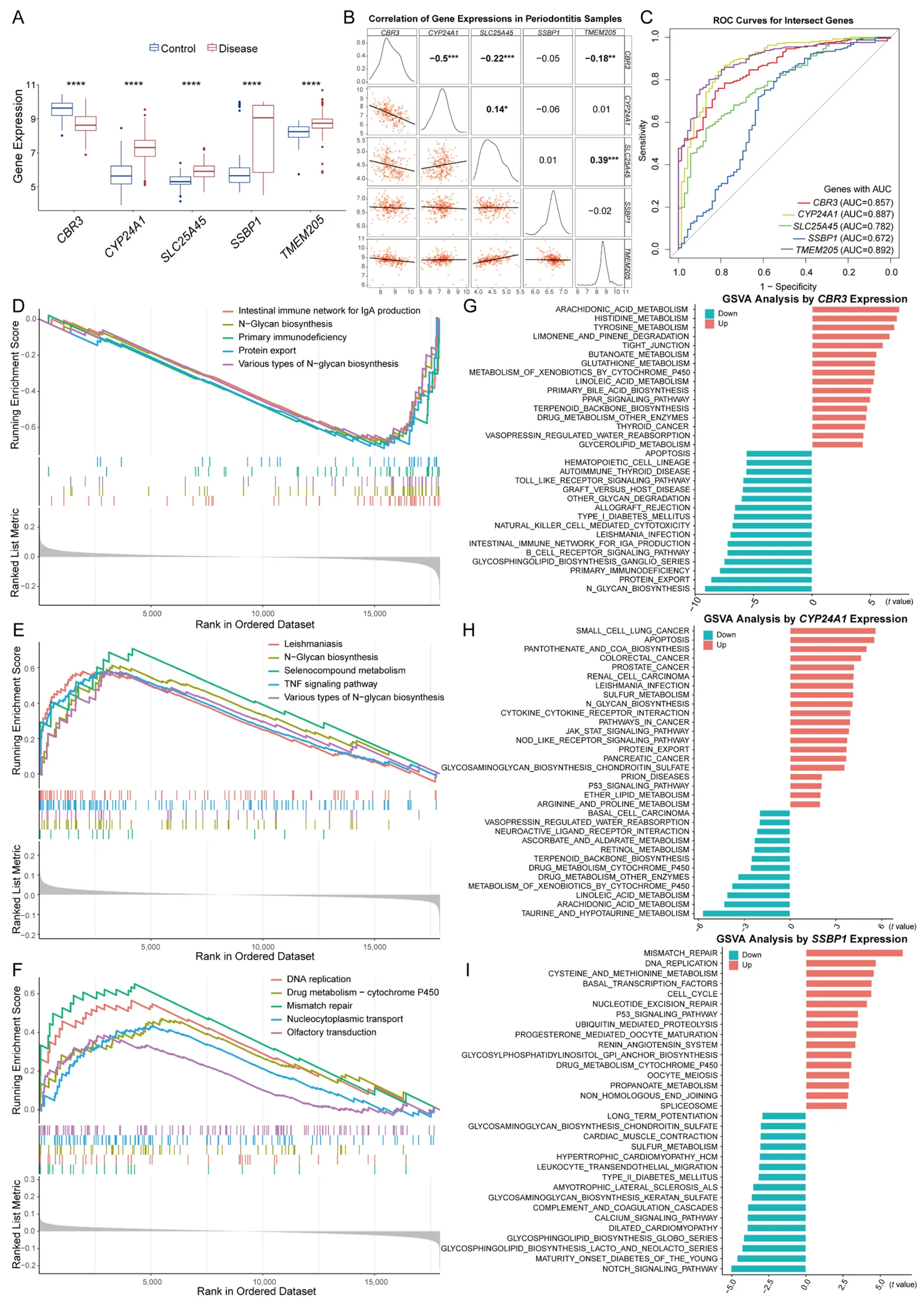
Expression patterns and pathway associations of the five selected genes. (**A**) Expression of *CBR3*, *CYP24A1*, *SLC25A45*, *SSBP1*, and *TMEM205* in affected and clinically unaffected gingival sites in GSE16134. (**B**) Pairwise correlations among the five genes. Orange dots represent individual samples, and black lines indicate linear regression fits. (**C**) Gene-level receiver operating characteristic curves in GSE16134. (**D**–**F**) Gene set enrichment analysis results for *CBR3*, *CYP24A1*, and *SSBP1*, respectively. (**G**–**I**) Corresponding gene set variation analyses for the same genes. AUC, area under the curve; FDR, false-discovery rate; GSEA, gene set enrichment analysis; GSVA, gene set variation analysis; NES, normalized enrichment score; ROC, receiver operating characteristic. * *p* < 0.05, ** *p* < 0.01, *** *p* < 0.001 and **** *p* < 0.0001.

In GSE16134, *CYP24A1*, *SLC25A45*, *SSBP1*, and *TMEM205* showed higher expression in affected gingival sites, whereas *CBR3* showed lower expression (all *p* < 0.05; Figure 4A). Pairwise correlations among the five genes were predominantly weak to moderate. The largest absolute correlation coefficients were observed between *CBR3* and *CYP24A1* (*r* = −0.50), *SLC25A45* and *TMEM205* (*r* = 0.39), and *CBR3* and *SLC25A45* (*r* = −0.22; Figure 4B).

Gene-level ROC analysis in GSE16134 yielded AUCs of 0.892 for *TMEM205*, 0.887 for *CYP24A1*, 0.857 for *CBR3*, 0.782 for *SLC25A45*, and 0.672 for *SSBP1* (Figure 4C). GSEA identified exploratory gene-specific pathway enrichment patterns involving pathways related to glycan biosynthesis and degradation, immune signaling, protein processing, and cellular metabolism (Figure 4D–F and Figure S3A,B). GSVA further demonstrated relationships between the expression of the selected genes and multiple metabolic, immune, inflammatory, and disease-related pathway signatures (Figure 4G–I and Figure S3C,D).

### 3.5. Immune-Cell Composition and Gene–Immune Associations

**Figure 5:**
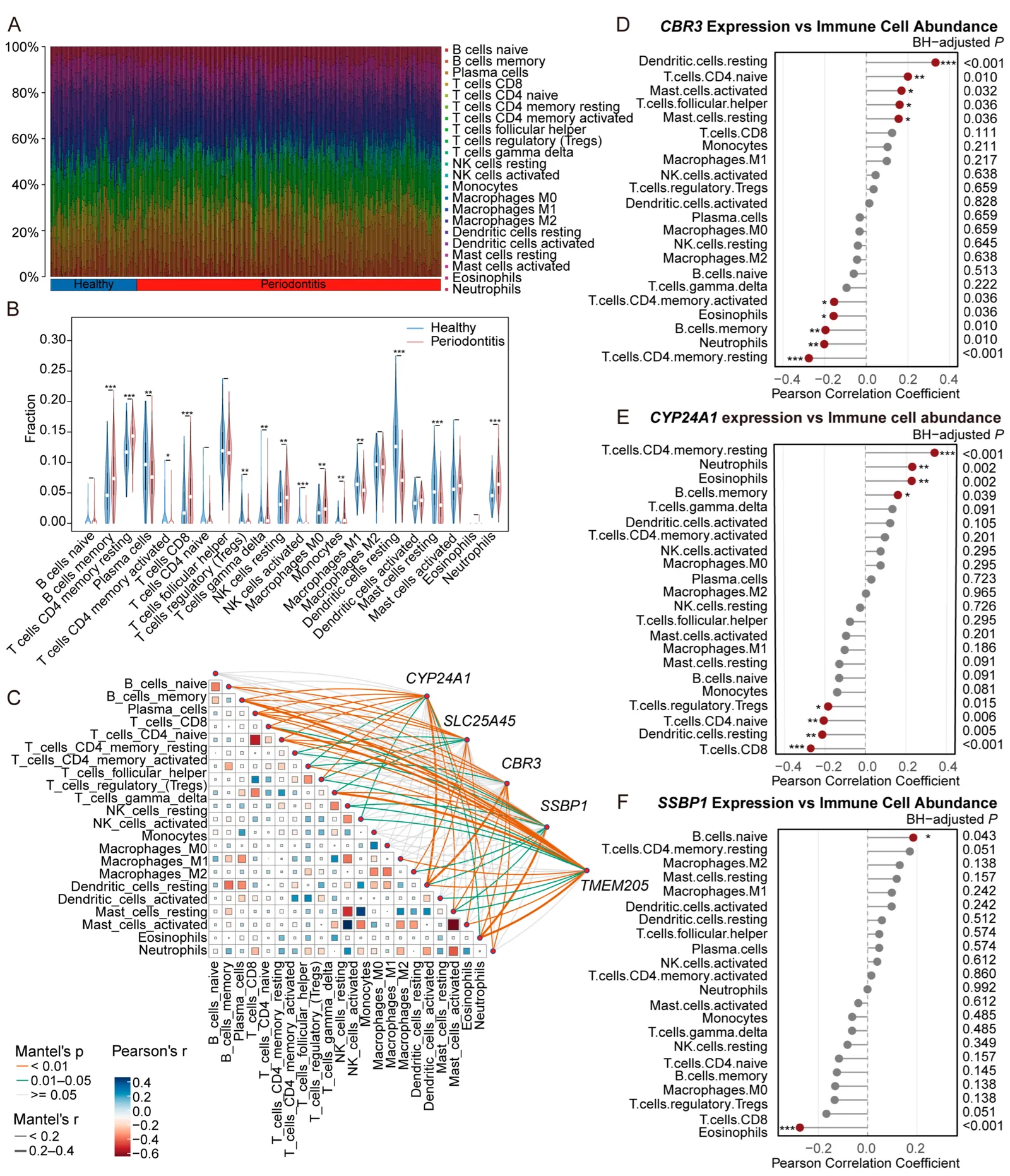
Inferred immune-cell composition and gene–immune associations in GSE16134. (**A**) Relative fractions of 22 immune-cell populations estimated using CIBERSORT. (**B**) Comparisons of inferred immune-cell fractions between affected and clinically unaffected gingival sites. (**C**) Correlation matrix of the inferred immune-cell fractions and the five selected genes. (**D**–**F**) Pearson correlations between inferred immune-cell fractions and the expression of *CBR3*, *CYP24A1*, and *SSBP1*, respectively, with Benjamini–Hochberg-adjusted *p* values shown on the right. Red dots represent items with statistically significant differences; gray dots represent non-significant results. * *p* < 0.05, ** *p* < 0.01, and *** *p* < 0.001. The Mantel’s r categories are distinguished by line thickness; the line representing Mantel’s r = 0.2–0.4 is twice as thick as the line representing Mantel’s r < 0.2.

CIBERSORT revealed significant differences in multiple inferred immune-cell fractions between affected and clinically unaffected gingival sites, involving B-cell, T-cell, natural-killer-cell, monocyte/macrophage, dendritic-cell, and neutrophil populations (Figure 5A,B). Correlation analysis further demonstrated distinct relationships between the five selected genes and the inferred immune-cell fractions (Figure 5C–F and Figure S4A,B). Among the strongest positive correlations, *CBR3* was associated with resting dendritic cells, *CYP24A1* with resting-memory CD4 T cells, *SLC25A45* with memory B cells, *SSBP1* with naive B cells, and *TMEM205* with plasma cells.

### 3.6. Establishment and Characterization of the Experimental Models

**Figure 6:**
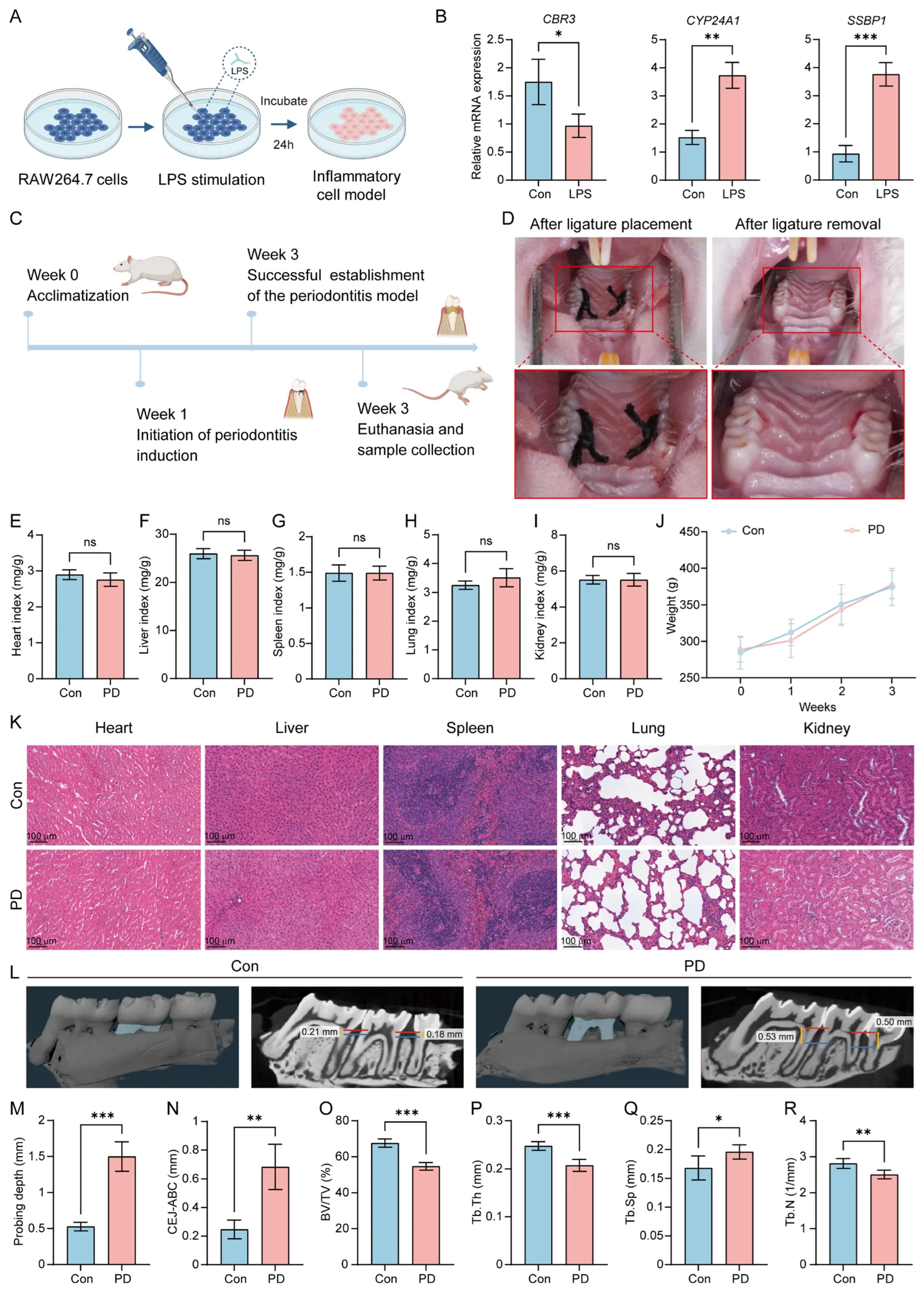
Establishment and characterization of the inflammatory macrophage and ligature-induced periodontitis models. (**A**) Schematic of the LPS-stimulation protocol for RAW 264.7 macrophages. Created in BioRender. Wu, S. (2026) https://BioRender.com/4syzf8g. (**B**) Relative mRNA expression of *Cbr3*, *Cyp24a1*, and *Ssbp1* in control and LPS-stimulated cells. Data were obtained from three independent biological experiments. (**C**) Timeline of the animal experiment. (**D**) Representative intraoral photographs of the maxillary second-molar region following ligature placement and removal. Red boxes indicate the regions shown at higher magnification. (**E**–**I**) Heart, liver, spleen, lung, and kidney indices. (**J**) Changes in body weight during the experimental period. (**K**) Representative hematoxylin and eosin staining of the major organs. (**L**) Representative three-dimensional micro-CT reconstructions and sagittal images of the maxillary second-molar region. In the micro-CT images, the blue region indicates the area of exposed root surface. In the sagittal images, the red line indicates the cementoenamel junction (CEJ), the blue line indicates the alveolar bone crest (ABC), and the yellow arrow indicates the CEJ–ABC distance. (**M**–**R**) Probing pocket depth, CEJ–ABC distance, BV/TV, Tb.Th, Tb.Sp, and Tb.N, respectively. For the animal experiments, *n* = 6 animals per group. Data are presented as the mean ± SD. ns, not significant; * *p* < 0.05, ** *p* < 0.01, and *** *p* < 0.001. BV/TV, bone volume fraction; CEJ–ABC, cementoenamel junction-to-alveolar bone crest; LPS, lipopolysaccharide; micro-CT, micro-computed tomography; Tb.N, trabecular number; Tb.Sp, trabecular separation; Tb.Th, trabecular thickness.

RAW 264.7 cells were exposed to LPS for 24 h to induce inflammatory activation (Figure 6A). Compared with untreated controls, LPS stimulation decreased *Cbr3* expression (*p* < 0.05) and increased *Cyp24a1* (*p* < 0.01), and *Ssbp1* (*p* < 0.001) expression (Figure 6B). The expression changes in *Cbr3*, *Cyp24a1*, and *Ssbp1* were directionally concordant with those observed in affected human gingival sites.

Ligature placement around the bilateral maxillary second molars produced visible gingival inflammation and an increased probing pocket depth (*p* < 0.001; Figure 6C,D,M). Body weight increased comparably in both groups throughout the experimental period (Figure 6J). No significant between-group differences were detected in the heart, liver, spleen, lung, or kidney indices, and histological examination revealed no apparent pathological alterations in these organs (Figure 6E–K).

Micro-CT analysis demonstrated greater alveolar bone loss in the periodontitis group (Figure 6L). Compared with control animals, ligature-treated rats showed an increased CEJ–ABC distance (*p* < 0.01) and trabecular separation (*p* < 0.05), together with decreased bone volume fraction (*p* < 0.001), trabecular thickness (*p* < 0.001), and trabecular number (*p* < 0.01; Figure 6N–R). These findings established the inflammatory and alveolar-bone-loss phenotypes of the ligature-induced periodontitis model.

### 3.7. Periodontal Histopathology and Expression of Selected Proteins

**Figure 7:**
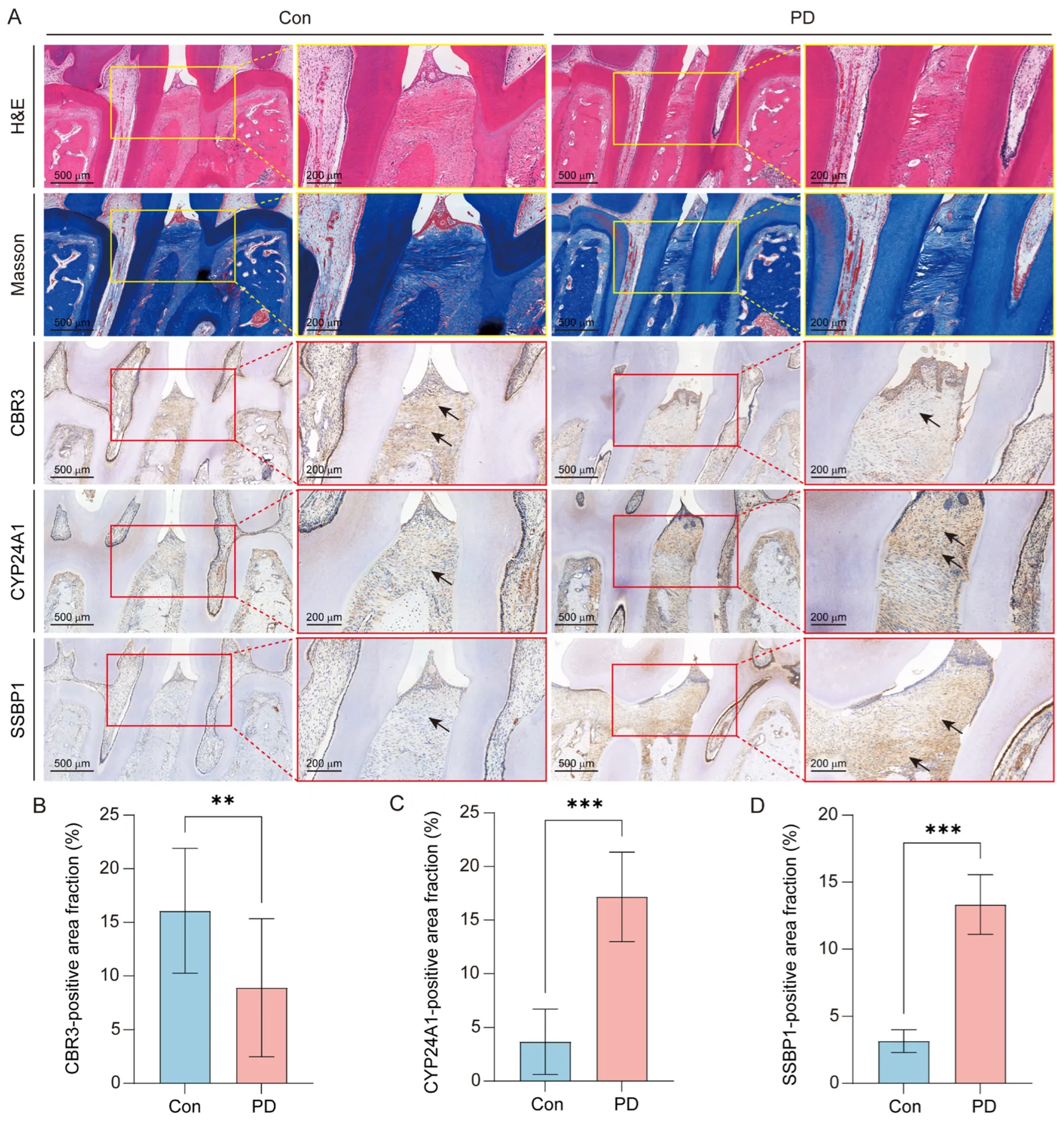
Periodontal histopathology and expression of selected proteins in the rat periodontitis model. (**A**) Representative hematoxylin and eosin staining, Masson’s trichrome staining, and immunohistochemical staining for CBR3, CYP24A1, and SSBP1 in periodontal tissues from the control and periodontitis groups. Arrows indicate positively stained areas. (**B**–**D**) Quantification of the CBR3-, CYP24A1-, and SSBP1-positive areas, respectively. *n* = 6 animals per group. Data are presented as the mean ± SD. ** *p* < 0.01 and *** *p* < 0.001.

Hematoxylin and eosin staining demonstrated periodontal tissue disruption in the periodontitis group, while Masson’s trichrome staining showed altered connective-tissue and collagen organization (Figure 7A). Immunohistochemical analysis revealed a lower CBR3-positive area in periodontitis tissues than in control tissues (*p* < 0.01; Figure 7B). In contrast, the CYP24A1-positive (*p* < 0.001), and SSBP1-positive (*p* < 0.001) areas were higher in the periodontitis group (Figure 7C–D). CBR3, CYP24A1, and SSBP1 therefore showed concordant directional changes across the human gingival transcriptomic dataset, LPS-stimulated macrophages, and rat periodontal tissues. SLC25A45 and TMEM205 were not examined in the experimental models.

To account for repeated gingival-site sampling within the same participant, we additionally performed a participant-aware sensitivity analysis using limma with duplicateCorrelation, which yielded consistent results (Figure S5).

## 4. Discussion

By integrating site-level gingival transcriptomics, co-expression network analysis, combinatorial machine learning, immune-cell deconvolution, and experimental expression assessment, this study identified a mitochondria-related transcriptional signature associated with periodontitis. Machine-learning analysis prioritized five genes—*CBR3*, *CYP24A1*, *SLC25A45*, *SSBP1*, and *TMEM205*—from an initial set of 25 candidates. Among them, *CBR3*, *CYP24A1*, and *SSBP1* showed the most consistent cross-system expression evidence, with concordant expression changes in affected human gingiva, LPS-stimulated macrophages, and rat periodontal tissues. Collectively, the selected genes encompass carbonyl and redox metabolism, vitamin D catabolism, mitochondrial metabolite transport, mtDNA maintenance, and membrane-associated processes. These findings suggest that periodontal inflammation is accompanied not by disruption of a single mitochondrial pathway, but by coordinated remodeling across several dimensions of mitochondrial biology. Recent transcriptomic and machine-learning studies have similarly linked periodontitis to alterations in mitochondria-related pathways, mitophagy, and the immune microenvironment. These studies provide broader computational support for the involvement of mitochondrial remodeling in periodontitis, although the reported molecular signatures and analytical strategies differ across datasets and studies [10,11,12,13,14].

Functional annotation provided exploratory support for this interpretation. The candidate genes were enriched in processes involving nucleotide and ATP metabolism, cellular respiration, respiratory-chain complex assembly, mitochondrial inner-membrane organization, and NAD(P)H-dependent oxidoreductase activity. Their associations with inferred immune-cell populations further suggested that mitochondria-related transcriptional changes were associated with the immune landscape of affected gingiva. Because mitochondria integrate redox balance, metabolite availability, bioenergetics, and innate immune signaling [4,5], these results suggest that mitochondria-related molecular changes may contribute to the metabolic and inflammatory features of periodontal tissues. Nevertheless, transcript abundance does not directly measure mitochondrial function, and the present findings should be interpreted as evidence of mitochondria-associated molecular remodeling rather than proof of mitochondrial dysfunction.

*CBR3* encodes an NADPH-dependent carbonyl reductase included in the MitoCarta3.0 mitochondrial inventory [22,34]. Its expression is regulated by NRF2 and can be modified by inflammatory stimuli in a cell-dependent manner [35,36]. The consistent reduction in CBR3 across human gingival tissues and both experimental systems is therefore notable. Chronic inflammation and oxidative stress generate reactive carbonyl species through lipid and carbohydrate oxidation, and reduced CBR3 expression could indicate impaired carbonyl-detoxification capacity or an altered NRF2-dependent stress response [37]. However, expression measurements alone cannot determine whether CBR3 deficiency promotes oxidative injury, represents a compensatory adaptation, or reflects changes in the cellular composition of diseased gingiva. Genetic manipulation of *CBR3*, together with measurements of reactive carbonyls, lipid peroxidation, NADPH availability, mitochondrial ROS, and respiratory activity, will be required to establish its functional contribution to periodontal inflammation.

*CYP24A1* encodes a mitochondrial cytochrome P450 enzyme that initiates the catabolism of both 25-hydroxyvitamin D and 1,25-dihydroxyvitamin D [38,39]. Vitamin D signaling contributes to epithelial antimicrobial defense, immune regulation, and bone homeostasis, and experimental evidence indicates that local vitamin D metabolism in gingival epithelium influences periodontal inflammation and alveolar bone loss [40,41]. The reproducible increase in *CYP24A1* across the three systems raises the possibility that enhanced local vitamin D inactivation attenuates vitamin D receptor-dependent protective responses in periodontal tissues. This hypothesis provides a biologically coherent connection between mitochondrial metabolism, host defense, and bone remodeling. Its verification will require direct measurement of vitamin D metabolites, CYP24A1 enzymatic activity, vitamin D receptor signaling, and the effects of CYP24A1 inhibition or genetic perturbation in periodontal cell and animal models.

Recent evidence has identified SLC25A45 as an inner-mitochondrial-membrane transporter for methylated amino acids, including dimethylarginine and trimethyllysine, with an essential role in de novo carnitine biosynthesis [42]. More broadly, members of the SLC25 family mediate the transport of diverse metabolites across the inner mitochondrial membrane and are essential for mitochondrial physiology and metabolic homeostasis [43]. Increased *SLC25A45* expression in affected gingiva may therefore reflect remodeling of mitochondrial amino-acid transport, carnitine availability, and fatty-acid metabolism. This possibility is particularly relevant to inflammatory tissues, in which metabolic substrate utilization and mitochondrial energy production are extensively reprogrammed. However, SLC25A45 was not directly examined in the experimental models. Direct assessment of SLC25A45, carnitine and acylcarnitine profiles, fatty-acid oxidation, and mitochondrial respiration would clarify the relevance of this metabolic axis.

*SSBP1* encodes mitochondrial single-stranded DNA-binding protein 1, a core component of the mitochondrial replisome that stabilizes single-stranded mtDNA and supports mtDNA replication, copy-number maintenance, and genome integrity [44,45]. Because periodontal inflammation is accompanied by oxidative stress and mitochondrial injury, increased demand for mtDNA maintenance may represent an important component of the mitochondrial stress response. The reproducible increase in *SSBP1* across affected human gingival tissues, LPS-stimulated RAW264.7 macrophages, and rat periodontal tissues is therefore notable. This consistent upregulation raises the possibility that SSBP1 is induced as an adaptive response to preserve mtDNA stability and sustain mitochondrial function during periodontal inflammation. Alternatively, increased *SSBP1* expression may reflect enhanced mtDNA replication stress or turnover and may not be sufficient to prevent mitochondrial dysfunction. Expression measurements alone cannot distinguish between these possibilities or establish whether SSBP1 has a protective or pathogenic role. Future studies should therefore combine genetic manipulation of *SSBP1* with assessments of mtDNA copy number, replication efficiency, oxidative mtDNA damage, cytosolic mtDNA release, mitochondrial ROS production, and respiratory activity to define its functional contribution to periodontal inflammation.

TMEM205 is included in MitoCarta3.0 as a mitochondrial membrane candidate, but its established literature has focused predominantly on cisplatin resistance [22,46]. Its role in mitochondrial physiology and periodontal inflammation remains poorly defined and requires direct localization and functional characterization in periodontal cells.

The immune-deconvolution results place the five genes within the broader immune remodeling of affected gingival tissues. Their correlations with selected immune-cell fractions suggest that mitochondria-related transcriptional changes are associated with the immune landscape of periodontitis. After multiple-testing correction using the Benjamini–Hochberg method, only selected gene–immune-cell associations remained statistically significant, and these relationships were interpreted as exploratory associations. Recent single-cell and spatial transcriptomic analyses have demonstrated that periodontitis-associated transcriptional changes arise from coordinated responses involving immune cells, keratinocytes, fibroblasts, endothelial cells, and spatially organized cellular niches [47]. Consequently, correlations derived from bulk-tissue deconvolution cannot establish whether a candidate gene is expressed by a particular immune population or directly regulates immune-cell recruitment or activation. Single-cell and spatial analyses, followed by cell-type-specific perturbation, will be necessary to localize the candidate genes and distinguish changes in cellular abundance from cell-intrinsic transcriptional regulation.

Several features of the transcriptomic and modeling design define the scope of the conclusions. GSE16134 comprises multiple affected and clinically unaffected sites from patients with established periodontitis; clinically unaffected sites therefore do not represent gingiva from periodontally healthy individuals [18,19,20]. Moreover, repeated gingival-site samples from the same participant may introduce within-subject correlation, which should be carefully considered in future studies using participant-level analytical strategies. GSE10334 partially overlaps the source cohort of GSE16134 [18,19,20], while performance in the two additional evaluation cohorts was more modest and imprecise. Because the original model-selection framework incorporated performance across multiple datasets, these datasets should be interpreted as evaluation cohorts. An additional independent validation using GSE223924, which was not involved in model selection or tuning, provided further support for the discriminatory ability of the selected model. The five-gene panel should therefore be interpreted as a hypothesis-generating molecular signature rather than a validated diagnostic classifier. Future studies should include larger, independent cohorts with prespecified modeling strategies and adequately powered participant-level validation to further evaluate its clinical applicability in accordance with TRIPOD + AI recommendations [17].

This study has several limitations. Although the experimental models provided cross-system support for selected expression changes, they did not establish causality. LPS-stimulated RAW 264.7 macrophages and ligature-induced rat periodontitis captured acute inflammatory activation and inflammation-associated alveolar bone loss, respectively, but neither fully recapitulated the polymicrobial and multicellular complexity of human periodontitis or established clinical relevance. Moreover, experimental validation was limited to expression-level changes and did not directly assess mitochondrial function. Future studies using human periodontal cell models, cell-resolved approaches, direct mitochondrial functional assays, and targeted manipulation of *CBR3*, *CYP24A1*, and *SSBP1* will be necessary to clarify the biological roles of these candidate genes.

Taken together, the results position mitochondrial state as a potentially important molecular context associated with metabolic, redox, and immune remodeling in periodontitis. The study does not establish that the identified genes initiate periodontal destruction; rather, it prioritizes specific and experimentally testable molecular axes associated with mitochondrial responses and the inflammatory periodontal microenvironment. This framework narrows the transition from broad descriptions of oxidative stress to specific mitochondria-related candidate pathways and markers that warrant mechanistic investigation and, ultimately, evaluated for translational relevance.

## 5. Conclusions

This study delineates a mitochondria-related transcriptional landscape associated with periodontitis and identifies *CBR3*, *CYP24A1*, and *SSBP1* as candidate molecular markers supported across human and experimental systems. By connecting periodontal inflammation with carbonyl and redox defense, vitamin D catabolism, mitochondrial metabolite transport, and mtDNA maintenance, the findings extend the mitochondrial framework of periodontitis beyond generalized oxidative stress. Participant-level validation, cell-resolved localization, and direct functional perturbation are now required to determine whether these candidates represent causal mediators, adaptive responses, or clinically informative markers. The resulting evidence provides a focused basis for future mechanistic investigation and clinical evaluation of mitochondria-related candidate markers in periodontitis.

## Figures and Tables

**Table 1:** Transcriptomic datasets included in the study.

Dataset	Platform	Samples Included in the Present Analysis	Analytical Role	Important Design Feature
GSE16134	Affymetrix HG-U133 Plus 2.0 array (Affymetrix, Santa Clara, CA, USA)	310 gingival sites from 120 participants (affected and clinically unaffected sites)	Discovery and model development	Multiple sites per participant
GSE10334	Affymetrix HG-U133 Plus 2.0 array	247 gingival sites from 90 participants (183 affected and 64 clinically unaffected sites)	Reproducibility assessment	Partially overlaps the GSE16134 source cohort; not independent validation
GSE173078	Illumina HiSeq 4000 RNA sequencing (Illumina, San Diego, CA, USA)	12 periodontitis and 12 healthy samples; 12 gingivitis samples excluded	Additional evaluation cohort	One sample per participant
GSE33774	Affymetrix Human Gene 1.0 ST array (Affymetrix, Santa Clara, CA, USA)	7 periodontitis and 8 healthy samples; 7 peri-implantitis samples excluded	Additional evaluation cohort	Small cross-platform cohort
GSE223924	Illumina NovaSeq 6000 RNA sequencing (Illumina, San Diego, CA, USA)	10 periodontitis and 10 healthy gingival tissue samples; 10 peri-implantitis samples excluded	Independent external validation	Independent RNA-seq cohort; one gingival tissue sample per participant

**Table 2:** Primer sequences used for RT-qPCR.

Gene	Forward Primer (5′–3′)	Reverse Primer (5′–3′)
*Cbr3*	CATCGACGACCCGCAGAG	GGTGTTGGGTCATCCATTCTAA
*Cyp24a1*	GGGCTGATGATCCTGGAAGG	GGACCATTTGTTCAGCTCGC
*Ssbp1*	ATGTGAAAAAGGGGGCTCGT	TGCCTTTTCTTTTGTCTGGTCAC
*Gapdh*	GTCAAGCTCATTTCCTGGTAT	TCTCTTGCTCAGTGTCCTTGC

**Table 3:** Diagnostic performance of the Stepglm[backward] + RF model in the discovery and external validation cohorts.

Cohort	AUC	Sensitivity (%)	Specificity (%)	PPV (%)	NPV (%)	Accuracy (%)
GSE16134	0.995	97.51	95.65	98.74	91.67	97.10
GSE10334	0.894	85.25	84.38	93.98	66.67	85.02
GSE33774	0.661	100.00	12.50	50.00	100.00	53.33
GSE173078	0.727	91.67	54.55	68.75	85.71	73.91
GSE223924	0.870	80.00	90.00	88.89	81.82	85.00

Note: Sensitivity, specificity, positive predictive value (PPV), negative predictive value (NPV), and accuracy were calculated using the optimal cutoff determined by the Youden index, with periodontitis defined as the positive class.

## Data Availability

The human transcriptomic datasets are publicly available from GEO (https://www.ncbi.nlm.nih.gov/geo/, accessed on 1 May 2025) under accession numbers GSE16134, GSE10334, GSE173078, GSE33774, and GSE223924. The MitoCarta3.0 inventory is available from the Broad Institute. Additional processed analysis files are available from the corresponding author on reasonable request.

## Supplementary Materials

The following supporting information can be downloaded at: https://www.mdpi.com/article/10.3390/biom16091369/s1, Figure S1: Gene Ontology enrichment analysis of DEGs and the WGCNA blue module; Figure S2: Calibration curves of the Stepglm[backward] + RF model across datasets; Figure S3: Pathway associations of *SLC25A45* and *TMEM205*; Figure S4: Gene–immune associations of *SLC25A45* and *TMEM205* in GSE16134; Figure S5: Participant-aware sensitivity analysis in GSE16134; Table S1: Gene Ontology enrichment analysis of DEGs; Table S2: Gene Ontology enrichment analysis of the WGCNA blue module.

## Abbreviations

AUC, area under the receiver operating characteristic curve; ABC, alveolar bone crest; ATP, adenosine triphosphate; BP, biological process; BV/TV, bone volume fraction; CC, cellular component; CEJ, cementoenamel junction; DEG, differentially expressed gene; DMEM, Dulbecco’s modified Eagle’s medium; FBS, fetal bovine serum; FDR, false-discovery rate; GEO, Gene Expression Omnibus; GO, Gene Ontology; GSEA, gene set enrichment analysis; GSVA, gene set variation analysis; IHC, immunohistochemistry; KEGG, Kyoto Encyclopedia of Genes and Genomes; LDA, linear discriminant analysis; LPS, lipopolysaccharide; MF, molecular function; MRG, mitochondria-related gene; NES, normalized enrichment score; PCA, principal-component analysis; PPD, probing pocket depth; RF, random forest; ROC, receiver operating characteristic; ROS, reactive oxygen species; RT-qPCR, reverse-transcription quantitative polymerase chain reaction; SD, standard deviation; SPF, specific pathogen-free; SVM, support vector machine; Tb.N, trabecular number; Tb.Sp, trabecular separation; Tb.Th, trabecular thickness; TOM, topological overlap matrix; WGCNA, weighted gene co-expression network analysis.
