# Supplementary material for: Integrative Transcriptomics Reveals a Mitochondria-Related Gene Signature in Periodontitis
Source: Biomolecules. 2026 Sep 20;16(9):1369. doi: 10.3390/biom16091369 (PMC13604177; doi:10.3390/biom16091369)
Supplement: Supplementary file 1 [file biomolecules-16-01369-s001.zip › biomolecules-4528633-supplementary final.pdf]

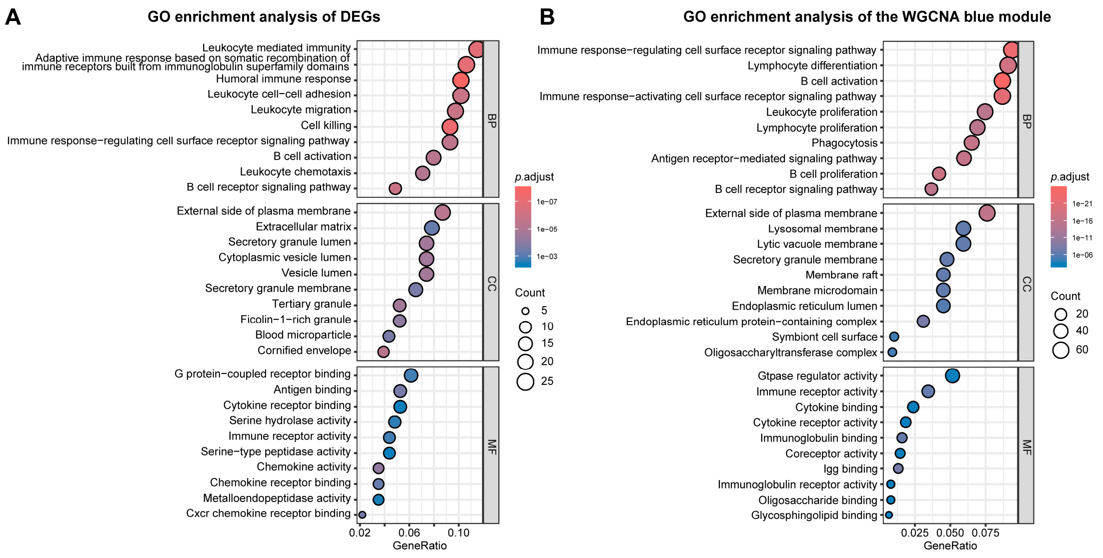

Figure S1. Gene Ontology enrichment analysis of DEGs and the WGCNA blue module. (A) GO enrichment analysis of all 264 DEGs identified in GSE16134. (B) GO enrichment analysis of all 911 genes in the WGCNA blue module. BP, biological process; CC, cellular component; MF, molecular function.

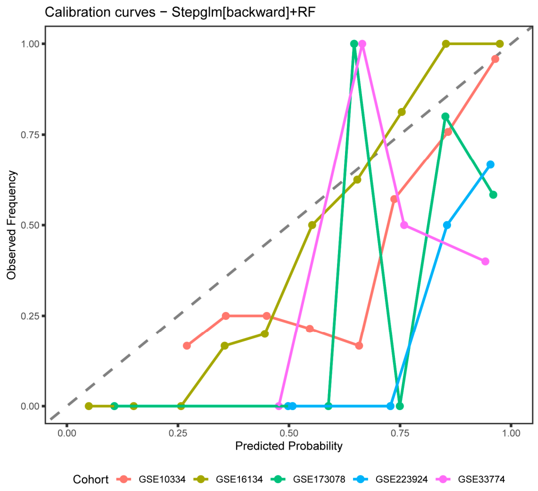

Figure S2. Calibration curves of the Stepglm[backward]+RF model across datasets.

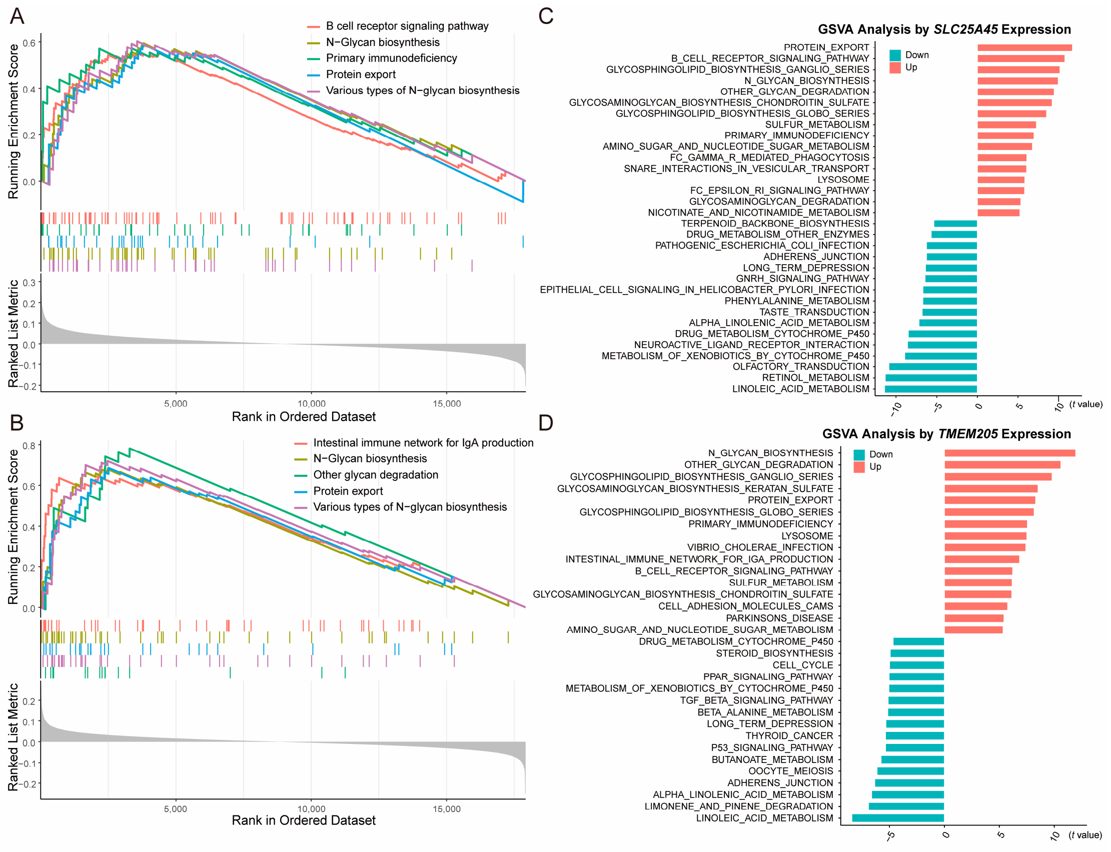

Figure S3. Pathway associations of *SLC25A45* and *TMEM205*. (A–B) Gene set enrichment analysis results for *SLC25A45* and *TMEM205*, respectively. (C–D) Corresponding gene set variation analyses for the same genes

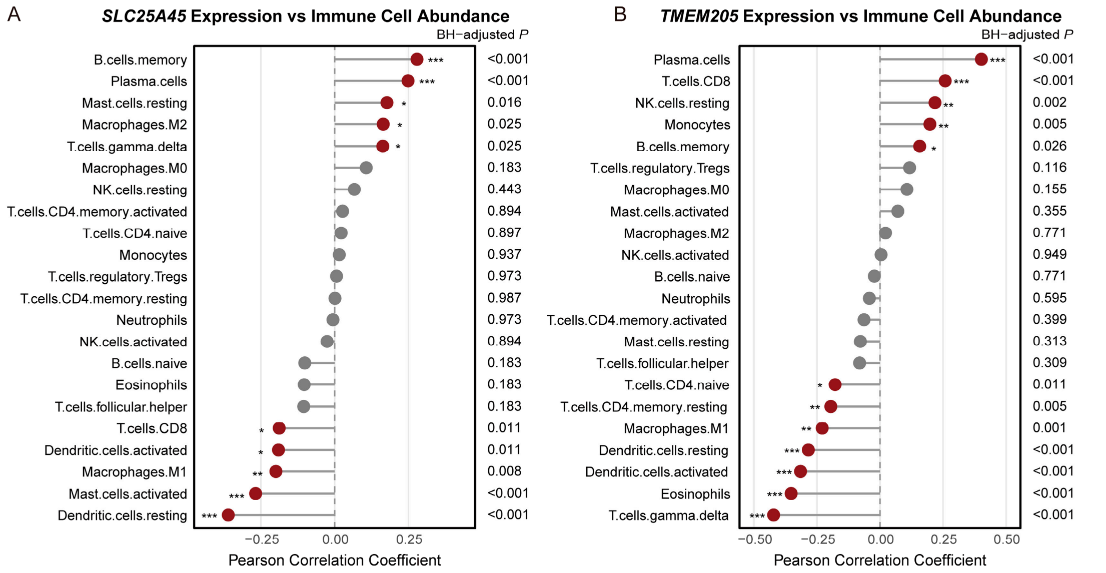

Figure S4. Gene–immune associations of *SLC25A45* and *TMEM205* in GSE16134. (A–B) Pearson correlations between inferred immune-cell fractions and the expression

of *SLC25A45* and *TMEM205*, respectively, with Benjamini–Hochberg-adjusted P values shown on the right.

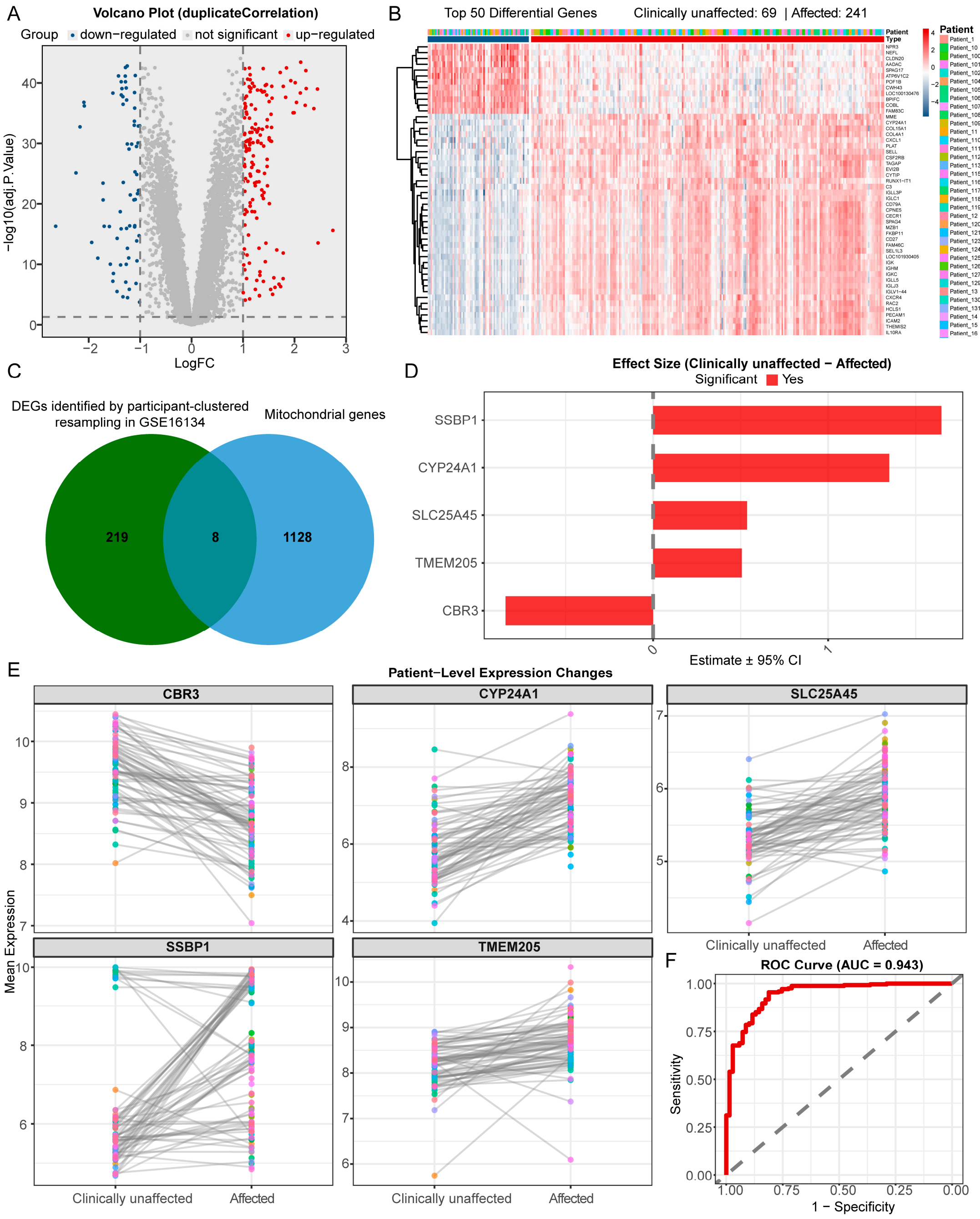

Figure S5. Participant-aware sensitivity analysis in GSE16134. (A) Volcano plot of differential expression analysis performed using limma with duplicateCorrelation to account for repeated gingival-site samples from the same participant. (B) Heatmap of the top 50 differentially expressed genes with participant-level annotation. (C) Intersection between differentially expressed genes identified in the participant-aware

analysis and MitoCarta3.0 mitochondrial genes. (D) Effect estimates and 95% confidence intervals for *CBR3*, *CYP24A1*, *SLC25A45*, *SSBP1*, and *TMEM205*. (E) Participant-level expression changes in the five selected genes between clinically unaffected and affected gingival sites. (F) Receiver operating characteristic curve of the participant-aware five-gene model. AUC, area under the curve; CI, confidence interval; ROC, receiver operating characteristic.
